# KDM6A facilitates Xist upregulation at the onset of X inactivation

**DOI:** 10.1186/s13293-024-00683-3

**Published:** 2025-01-03

**Authors:** Josephine Lin, Jinli Zhang, Li Ma, He Fang, Rui Ma, Camille Groneck, Galina N. Filippova, Xinxian Deng, Chizuru Kinoshita, Jessica E. Young, Wenxiu Ma, Christine M. Disteche, Joel B. Berletch

**Affiliations:** 1 Department of Laboratory Medicine and Pathology, School of Medicine, University of Washington, Seattle, WA 98195 USA; 2 Department of Statistics, University of California Riverside, Riverside, CA 92521 USA; 3 Department of Microbiology, Immunology & Cell Biology, University of West Virginia, Morgantown, WV 26506 USA; 4 Department of Biochemistry, University of California Riverside, Riverside, CA 92521 USA; 5 Department of Medicine, School of Medicine, University of Washington, Seattle, WA 98195 USA

**Keywords:** Allele-specific, Epigenetics, X inactivation, Histone methylation, Dosage compensation, Escape

## Abstract

**Background:**

X chromosome inactivation (XCI) is a female-specific process in which one X chromosome is silenced to balance X-linked gene expression between the sexes. XCI is initiated in early development by upregulation of the lncRNA *Xist* on the future inactive X (Xi). A subset of X-linked genes escape silencing and thus have higher expression in females, suggesting female-specific functions. One of these genes is the highly conserved gene *Kdm6*a, which encodes a histone demethylase that removes methyl groups at H3K27 to facilitate gene expression. *KDM6A* mutations have been implicated in congenital disorders such as Kabuki Syndrome, as well as in sex differences in development and cancer.

**Methods:**

*Kdm6a* was knocked out (KO) using CRISPR/Cas9 gene editing in hybrid female mouse embryonic stem (ES) cells derived either from a 129 × *Mus castaneus* (*cast*) cross or a BL6 x *cast* cross. In one of the lines a transcriptional stop signal inserted in *Tsix* results in completely skewed X silencing upon differentiation. The effects of both homozygous and heterozygous *Kdm6a* KO on *Xist* expression during the onset of XCI were measured by RT-PCR and RNA-FISH. Changes in gene expression and in H3K27me3 enrichment were investigated using allele-specific RNA-seq and Cut&Run, respectively. KDM6A binding to the *Xist* gene was characterized by Cut&Run.

**Results:**

We observed impaired upregulation of *Xist* and reduced coating of the Xi during early stages of differentiation in *Kdm6a* KO cells, both homozygous and heterozygous, suggesting a threshold effect of KDM6A. This was associated with aberrant overexpression of genes from the Xi after differentiation, indicating loss of X inactivation potency. Consistent with KDM6A having a direct role in *Xist* regulation, we found that the histone demethylase binds to the *Xist* promoter and KO cells show an increase in H3K27me3 at *Xist*, consistent with reduced expression.

**Conclusions:**

These results reveal a novel female-specific role for the X-linked histone demethylase, KDM6A in the initiation of XCI through histone demethylase-dependent activation of *Xist* during early differentiation.

**Plain language summary:**

X chromosome inactivation is a female-specific mechanism that evolved to balance sex-linked gene dosage between females (XX) and males (XY) by silencing one X chromosome in females. X inactivation begins with the upregulation of the long noncoding RNA *Xist* on the future inactive X chromosome. While most genes become silenced on the inactive X chromosome some genes escape inactivation and thus have higher expression in females compared to males, suggesting that escape genes may have female-specific functions. One such gene encodes the histone demethylase KDM6A which function is to turn on gene expression by removing repressive histone modifications. In this study, we investigated the role of KDM6A in the regulation of *Xist* expression during the onset of X inactivation. We found that KDM6A binds to the *Xist* gene to remove repressive histone marks and facilitate its expression in early development. Indeed, depletion of KDM6A prevents upregulation of *Xist* due to abnormal persistence of repressive histone modifications. In turn, this results in aberrant overexpression of genes from the inactive X chromosome. Our findings point to a novel mechanism of *Xist* regulation during the initiation of X inactivation, which may lead to new avenues of treatment to alleviate congenital disorders such as Kabuki syndrome and sex-biased immune disorders where X-linked gene dosage is perturbed.

**Supplementary Information:**

The online version contains supplementary material available at 10.1186/s13293-024-00683-3.

## Highlights

- *Kdm6a* knockout prevents upregulation of the lncRNA *Xist* during differentiation of female stem cells, suggesting that KDM6A plays a threshold-dependent role in facilitating *Xist* expression during the onset of X chromosome inactivation, a female-specific phenomenon.
- X chromosome inactivation efficacy is markedly reduced in cells with *Kdm6a* knockout, leading to increased gene expression from the Xi.
- KDM6A binding at the *Xist* gene during the onset of X inactivation is necessary for the removal of H3K27 methylation, revealing a direct role in regulating *Xist* expression via H3K27 demethylation-dependent mechanisms.

**Supplementary Information:**

The online version contains supplementary material available at 10.1186/s13293-024-00683-3.

## Introduction

In mammalian females X chromosome inactivation (XCI) serves to balance X-linked gene expression between males (XY) and females (XX) [1, 2]. XCI is a robust silencing mechanism characterized by several chromosome-wide epigenetic changes on the inactive X (Xi), including histone de-acetylation, ubiquitination of H2AK119, methylation of H3K27 and H3K9, DNA methylation at CpG islands, and chromatin condensation. XCI is initiated at early stages of development by upregulation of the long non-coding RNA (lncRNA) *Xist* from the future Xi. *Xist* RNA coats the Xi and recruits proteins that implement epigenetic changes for silencing. This process can be modeled in mouse embryonic stem (ES) cells, which demonstrate extensive silencing by day 4–7 of differentiation [3, 4].

A subset of X-linked genes escape XCI and remain expressed from the Xi, albeit at a lower level than from the active X chromosome (Xa), resulting in higher expression in females compared to males [5, 6]. XCI and escape are processes unique to females, suggesting that factors involved in these processes such as *Xist* and other regulatory elements that influence the structure and epigenetic features of the Xi may be partly controlled by genes with female-biased expression [7]. One of these genes is *Kdm6a*, a highly conserved gene that escapes XCI in all mammalian species tested [8, 9]. The main function of KDM6A is to facilitate gene expression during differentiation and development by resolution of bivalent domains through demethylation of H3K27me3 [10–13]. Additionally, KDM6A plays a role in allelic gene regulation, where it preferentially targets maternal alleles of autosomal genes to increase expression [14]. Interestingly, KDM6A has a Y-encoded homolog (UTY) that has little or no demethylase activity, suggesting that KDM6A may have female-specific roles that depend on its demethylase function [15–17].

Here, we show that KDM6A plays a role in *Xist* upregulation during the earliest stages of XCI. We find that *Kdm6a* KO results in decreased levels of *Xist* RNA and in reduced formation of an *Xist* cloud during ES cell differentiation. This causes impaired silencing of genes from the Xi. A direct association between H3K27me3 demethylation by KDM6A and *Xist* expression is supported by findings that KDM6A binds to the *Xist* promoter region during ES cell differentiation, and that *Kdm6a* KO results in an increase of H3K27me3 levels along the *Xist* gene. Thus, the female-biased expression of *Kdm6a* via escape from XCI may have evolved as a mechanism that contributes to XCI by regulating *Xist*.

## Results

### Generation of *Kdm6a* KO ES cells

To study the potential role of KDM6A at the onset of XCI, we used CRISPR/Cas9 to knockout (KO) *Kdm6a* in hybrid female mouse ES cells derived from a 129 x *Mus castaneus* (*cast*) cross in which alleles can be distinguished by SNPs (Additional File 1: Figure S1A). In order to examine changes specifically on the Xi we used *Tsix-*stop cells (donated by J. Gribnau, Erasmus MC) in which a transcriptional stop signal is inserted onto the 129 allele of *Tsix*, resulting in completely skewed silencing of the 129 X chromosome upon differentiation [18]. Stable *Kdm6a* KO *Tsix-*stop ES cell clones with a heterozygous or homozygous deletion of part of exon 2 through part of exon 4 (wt/ΔE or ΔE/ΔE, respectively) were generated (Additional File 1: Figure S1A) [14]. PCR and Sanger sequencing verified homozygous editing of *Kdm6a* in two KO clones (*Tsix-Kdm6a*^*ΔE/ΔE17*^ and *Tsix-Kdm6a*^*ΔE/ΔE21*^) and heterozygous editing in one KO clone (*Tsix-Kdm6a*^*wt/ΔE4*^). Despite residual *Kdm6a* expression in the homozygous KO clones, western blot analysis showed no evidence of KDM6A protein [14, 15]. We also isolated control *Tsix-*stop ES cell clones (*Tsix*-cln1, *Tsix*-cln2; hereafter called CRISPR controls), which were subject to the CRISPR/Cas9 treatment but did not exhibit a deletion. In a separate experiment, *Kdm6a* homozygous editing was done by deleting the promoter in another hybrid female mouse ES cell line called E8 (donated by J. Gribnau, Erasmus MC) to derive clone E8-*Kdm6a*^*ΔP/ΔP13*^ (Additional File 1: Figure S1A). The E8 cell line derived from a C57BL/6J (BL6) x *cast* cross undergoes random XCI with partial skewing due to a strong *Xce* allele that results in a ~2:1 inactivation of the BL6:*cast* X chromosome upon differentiation [4, 19]. A summary of the clones used in this study can be found in Additional File 2: Table S1.

### *Kdm6a* KO results in expression changes of genes involved in germ layer development after XCI occurs

**Fig. 1:**
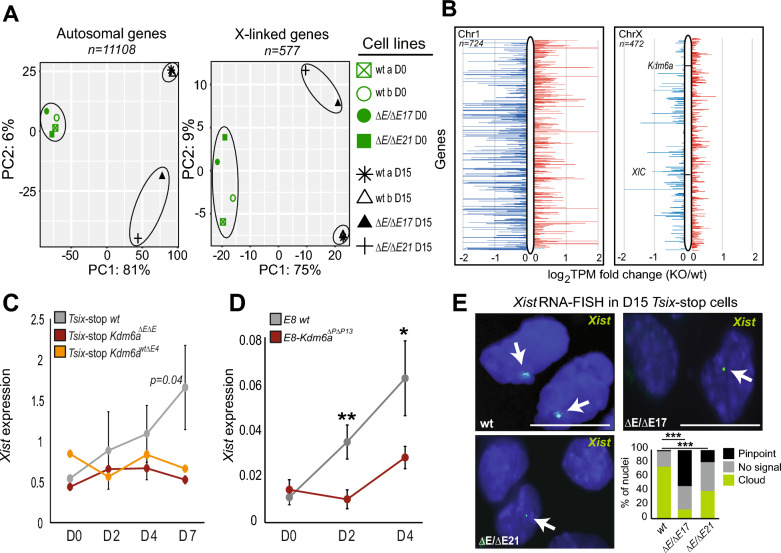
*Kdm6a* KO results in *Xist* downregulation. **A** Principal component analysis (PCA) based on expression of autosomal or X-linked genes in two *Tsix*-stop wt clones and two *Kdm6a* KO clones (*Tsix-Kdm6a*^*ΔEΔE17*^ and *Tsix-Kdm6a*^*ΔEΔE21*^) before (D0) and after differentiation (D15). Only genes with a minimum of 2.5 CPM in 4 libraries were considered. PCA shows separation of clones based on *Kdm6a* KO status mainly after differentiation. PCA figures were generated by iDEP.95. **B** Plots of diploid gene expression from a representative autosome (Chr1, left) with similar gene content as the X, and from the X chromosome (right) in *Tsix*-stop wt and KO clones (average for *Tsix-Kdm6a*^*ΔEΔE17*^ and *Tsix-Kdm6a*^*ΔEΔE21*^) at D15. *Kdm6a* and the XIC are highlighted. Log_2_ TPM fold change (KO/wt) is shown. Genes with a decrease of expression in KO versus wt are in blue, and with an increase in red. **C** qRT-PCR of average *Xist* expression normalized to *Actinβ* during ES differentiation in three *Tsix*-stop wt samples, two *Kdm6a*^*ΔEΔE*^ KO clones, and one *Kdm6a*^*ΔwtΔE*^ clone. p-value is derived from comparing wt to *Kdm6a*^*ΔEΔE*^ biological replicates. Divergence of *Xist* expression between wt and KO cells begins at D2 and is largest at D7 (p = 0.04). The *Kdm6a*^*wt/ΔE4*^ heterozygous KO clone behaves similarly to the homozygous clones. **D** qRT-PCR of *Xist* expression in E8 wt and KO clone (E8-*Kdm6a*^*ΔPΔP13*^). Expression is normalized to *Actinβ*. *Xist* is significantly lower at D2 and D4 in KO cells. P-values derived from two technical replicates of wt and KO cells (**p < 0.01;*p < 0.05). **E** RNA-FISH with a probe specific for *Xist* RNA labeled in green in *Tsix*-stop wt and KO clones (*Tsix-Kdm6a*^*ΔEΔE17*^ and *Tsix-Kdm6a*^*ΔEΔE21*^). Examples of nuclei are shown together with histograms for quantification of signal type (pinpoint, no signal or cloud). *Xist* clouds are present in 74% of D15 wt cells, but significantly decrease in KO clones (cloud compared to no signal; ***p < 0.00001 for T*six-Kdm6a*^*ΔEΔE17*^ and < 0.0001 for *Tsix-Kdm6a*^*ΔEΔE21*^ fisher’s exact test), while the frequency of pinpoint *Xist* signals increases in KO clones (pinpoint in wt compared to pinpoint in KO; ***p < 0.00001 for *Tsix-Kdm6a*^*ΔEΔE17*^ and *Tsix-Kdm6a*^*ΔEΔE21*^ fisher’s exact test). Nuclei are counterstained with Hoechst 33,342. Scale bar = 10 µm

RNA-seq analyses were done to compare gene expression between *Tsix-*stop wild-type (wt) and *Kdm6a* KO (*Tsix-Kdm6a*^*ΔE/ΔE17*^ and *Tsix-Kdm6a*^*ΔE/ΔE21*^) clones before day 0 (thereafter abbreviated D0) and after (D15) differentiation of ES cells into embryoid bodies (EB). For *Tsix-*stop cells, principal component analysis (PCA) based on either autosomal or X-linked gene expression shows little separation between wt and KO clones at D0, but a clear separation at D15, indicating that the effects of *Kdm6a* KO are most pronounced after differentiation (Figure 1A). Consistent with PCA clustering, we found only 624 differentially expressed genes (DEGs) between wt and KO cells at D0, while there were 886 DEGs at D15 (≥2 fold change; FDR ≤0.1) (Additional File 1: Figure S1B; Additional File 3: Table S2). These findings are in agreement with KDM6A’s dispensable role in undifferentiated ES cells versus its multiple roles in regulation of genes involved in development and differentiation [11, 14, 20]. Indeed, among the DEGs found at D15 (a time point after XCI has occurred) there is a subset of genes involved in germ layer development, including genes expressed in all three germ layers, which are downregulated in *Tsix-*stop *Kdm6a* KO cells at D15, but not at D0 (Additional File 1: Figure S1C; Additional File 4: Table S3). Of the germ layer genes with decreased expression in *Tsix*-stop KO cells, 19 were expressed (>1 TPM) at D2 and 18 were expressed at D4 of differentiation in E8-*Kdm6a* wt cells. There was no clear pattern of downregulation of these genes at either time point following *Kdm6a* KO, strongly suggesting that germ layer gene downregulation occurred after XCI (Additional File 1: Figure S1C; Additional File 4: Table S3). In line with KDM6A’s role in facilitating gene expression by removing the repressive mark H3K27me3, we observed an overall decrease in autosomal gene expression (Figure 1B). In contrast, 217/472 (46%) of X-linked genes assayed showed increased expression (>1.25 TPM fold change) in *Tsix*-stop *Kdm6a* KO (average of clones *Tsix-Kdm6a*^*ΔE/ΔE17*^ and *Tsix-Kdm6a*^*ΔE/ΔE21*^) compared to wt at D15 (Figure 1B).

### *Xist* expression and coating are impaired during early differentiation following *Kdm6a* KO

One of the earliest events at the onset of XCI is the upregulation of *Xist* [1, 2]. Therefore, we investigated changes in the dynamics of *Xist* expression during early mouse ES cell differentiation at D0, D2, D4, D7, and D15 following *Kdm6a* KO. Compared to *Tsix-*stop wt and CRISPR-control clones, *Kdm6a* KO clones (*Tsix-Kdm6a*^*ΔE/ΔE17*^ and *Tsix-Kdm6a*^*ΔE/ΔE21*^) have significantly reduced levels of *Xist* expression beginning at D2 of differentiation when *Xist* upregulation normally starts (Figures 1C and Additional File 5: Figure S2A). Importantly, this finding was confirmed in *Kdm6a* KO clone (E8-*Kdm6a*^*ΔP/ΔP13*^) derived from an independent ES line E8 (Figure 1D). Thus, we infer that KDM6A contributes to the activation of *Xist* in early differentiation beginning prior to D2 and increasing at D4 and D7 (Figures 1B and Additional File 5: Figure S2B). Analysis in *Tsix*-stop cells with heterozygous deletion of *Kdm6a (Tsix-Kdm6a *^*wt/ΔE4*^) revealed a similar effect on *Xist* by D7, suggesting that expression of *Kdm6a* from both alleles is needed for activation of *Xist* (Figure 1C). The lower expression of *Xist* in *Kdm6a* KO cells does not appear to be due to changes in levels of several of the known *Xist* repressors (*Tsix*, *Nanog, Pou5f1, Sox2, Rex1)* or activators (*Yy1, Rlim*) as these were not differentially expressed between *Tsix*-stop wt and KO cells at D0 (Additional File 5: Figure S2C) [21]. *Klf4*, which is known to repress *Xist* via activation of *Tsix* [22], did show an increase in *Kdm6a* KO cells at D0, but *Tsix* expression was consistently lower in KO cells at D2, reaching levels similar to those in wt only at D4, suggesting that the increase in *Klf4* did not affect *Tsix* expression at this stage (Additional File 5: Figure S2D and E; Additional File 3: Table S2). Note that despite a small increase in *Sox2* expression in *Kdm6a* KO cells, this gene was not called as a DEG by Deseq2 (Additional File 5: Figure S2C; Additional File 3: Table S2). Previous studies have shown aberrantly increased expression of *Xis*t with *Tsix* inhibition in undifferentiated ES cells grown in media with serum [23, 24]. However, we verified that the *Tsix-*stop ES cells used here maintain a low level of *Xist* expression at D0 (~2 TPM) similar to levels observed in previous studies despite the presence of serum in the media (Additional File 5: Figure S2F) [3, 14].

To determine whether *Kdm6a* KO affects the formation of an *Xist* cloud we performed RNA-FISH with a probe that targets *Xist* in differentiated *Tsix-*stop *Kdm6a* KO and wt cells at D15. The percentage of nuclei with *Xist* clouds sharply decreased after KO, with only 18% and 38% of nuclei with an *Xist* cloud in clones *Tsix-Kdm6a*^*ΔE/ΔE17*^ and *Tsix-Kdm6a*^*ΔE/ΔE21*^, respectively, versus 74% of wt nuclei (Figure 1E). Consistent with reduced *Xist* expression in *Kdm6a* KO clones, nuclei with a small pinpoint *Xist* signal rather than a cloud were prevalent in these clones (41% and 23% of nuclei in clones *Tsix*-*Kdm6a*^*ΔE/ΔE17*^ and *Tsix-Kdm6a*^*ΔE/ΔE21*^, respectively) compared to 4% in wt nuclei (Figure 1E). Loss of an X chromosome during cell culture is a common occurrence in female mouse ES cells and would confound our analysis of X-linked gene expression. To address this possibility DNA-FISH using probes targeting X chromosome-specific regions was done to verify that minor X chromosome loss occurred in any condition tested (Additional File 6: Figure S3).

Together, these results indicate that KDM6A contributes to upregulation of *Xist* expression in a threshold dependent manner, and to *Xist* coating of the Xi during early mouse ES cell differentiation.

### Aberrantly increased gene expression from the Xi in differentiated *Kdm6a* KO cells

**Fig. 2:**
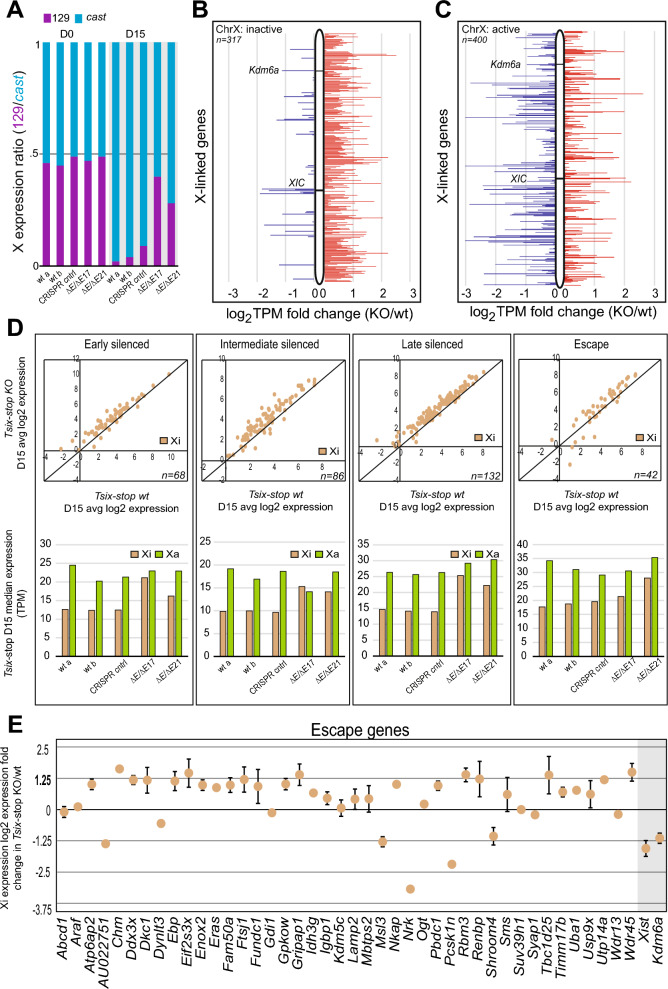
*Kdm6a* KO leads to increased gene expression from the Xi in differentiated cells. **A** Allelic X-linked gene expression ratios (129:cast) in *Tsix*-stop wt, CRISPR control, and KO cells (*Tsix-Kdm6a*^*ΔEΔE17*^ and *Tsix-Kdm6a*^*ΔEΔE21*^) at D0 and D15 of differentiation. Ratios between expressed X-linked genes (> 1TPM) are shown. There is increased gene expression from the 129 X-chromosome (Xi) following *Kdm6a* KO differentiation. **B, C** Plots of expression from the Xi **(B)** and the Xa **(C)** (average for *Tsix-Kdm6a*^*ΔEΔE17*^ and *Tsix-Kdm6a*^*ΔEΔE21*^) at D15. *Kdm6a* and the XIC are highlighted. Log_2_ TPM fold change (KO/wt) is shown. Genes with a decrease of expression in KO versus wt are in blue, and with an increase in red. Gene expression from the Xi is selectively increased following KO. **D** Top, scatter plots of average log_2_ expression between *Tsix-*stop wt and KO clones (average for *Tsix-Kdm6a*^*ΔEΔE17*^ and *Tsix-Kdm6a*^*ΔEΔE21*^) at D15 for X-linked genes categorized as those silenced early, silenced at an intermediate time, silenced late, and not silenced (escape) during XCI [3, 25]. KO clones show higher expression of genes in each category compared to wt. n indicates the number of genes in each category. Bottom, histograms of median allelic expression of genes silenced at different times during XCI and of genes that escape XCI is increased in *Tsix-*stop KO clones compared to wt and CRISPR controls for all categories, while there is little change in expression from the Xa. n indicates the number of genes in each category. **E** Expression fold changes of individual escape genes from the Xi in *Tsix-*stop KO clones (average for *Tsix-Kdm6a*^*ΔEΔE17*^ and *Tsix-Kdm6a*^*ΔEΔE21*^) compared to wt at D15. Most genes show increased expression

We postulated that reduced *Xist* upregulation and *Xist* coating in *Kdm6a* KO cells may lead to impaired XCI in these cells. Allelic analyses of X-linked gene expression in *Tsix*-stop cells show that the increase in expression following *Kdm6a* KO is mainly due to increased expression specifically from the Xi where 76% (242/317) of assayed genes show a ≥ 1.25 TPM fold increase (Figure 2A, B; Additional File 8: Table S4). In contrast, there is no clear directionality for dysregulated genes on the Xa (54% up and 46% downregulated; ≥ 1.25 TPM fold cutoff) (Figure 2C). In addition, we confirmed increased X-linked gene expression in an independent *Kdm6a* KO clone (E8-*Kdm6a*^*ΔP/ΔP13*^) where 42% (208/491) of X-linked genes show a ≥ 1.25 TPM fold increased expression at D4 of differentiation (Additional File 7: Figure S4; Additional File 8: Table S4). In E8 cells, where XCI is largely random, allelic analyses show increased X-linked gene expression following *Kdm6a* KO coming from the BL6 and *cast* X (Additional File 7: Figure S4A, B). However, gene expression is slightly more elevated from the BL6 X, suggestive of a specific effect of *Kdm6a* KO on the Xi, which would tend to be the BL6 X due to partial XCI skewing in E8 cells (Additional File 7: Figure S4C, D). Thus, failure to upregulate *Xist* leads to increase X-linked gene expression in differentiated cells. Considering autosomal genes, *Kdm6a* KO resulted in a majority of dysregulated genes with decreased expression (84% and 60% of dysregulated genes in *Tsix*-stop *Kdm6a* KO clones and in E8-*Kdm6a*^*ΔP/ΔP13*^, respectively), consistent with the role of KDM6A in facilitating gene expression through removal of H3K27me3 (Additional File 3: Table S2).

Next, we grouped X-linked genes in four defined temporal clusters, early, intermediate, late, and not silenced (or escape), based on their previously reported timing of silencing during differentiation [3, 25]. All gene clusters show a trend of increased expression from the Xi in *Tsix-*stop *Kdm6a* KO cells compared to wt or CRISPR controls (Figures 2D, E; Additional File 9: Table S5). Interestingly, the expression of most escape genes is also increased after *Kdm6a* KO (Figure 2D, E). For all clusters the increase is usually less than ~ 1.25 fold, with few escape genes showing expression changes > 1.25 fold in KO versus wt ES cells. Few escape genes show a decrease in expression and include *Xist* and *Kdm6a*, as expected (Figure 2E).

Taken together, these results indicate a reduced potency of XCI in differentiated *Kdm6a* KO cells, which implicates KDM6A, at least in part, as a regulator of X-linked gene expression dosage via upregulation of *Xist* during the onset of XCI.

### *Kdm6a* KO results in dysregulated H3K27me3 levels on the X chromosome

**Fig. 3:**
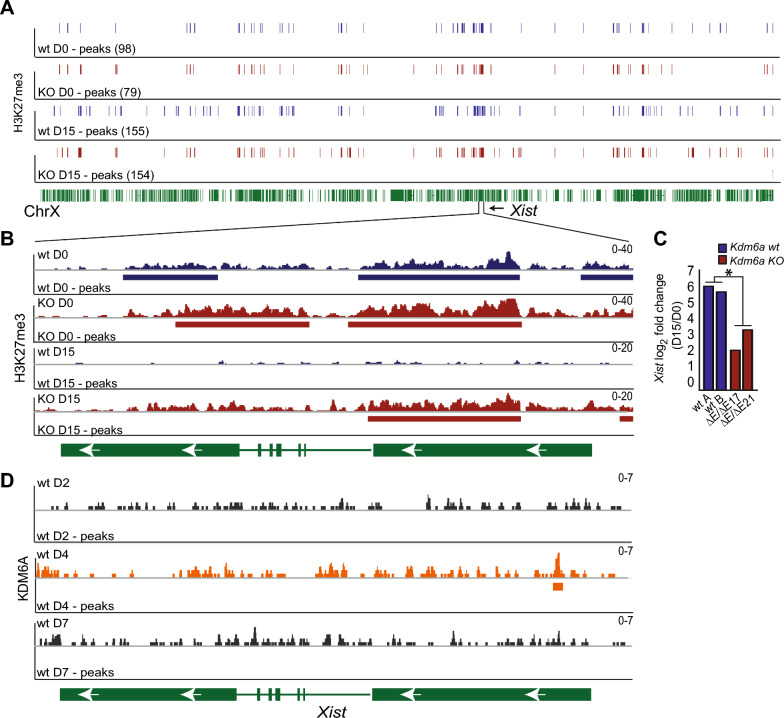
KDM6A binds to the promoter of *Xist* and *Kdm6a* KO results in increased H3K27me3. **A** IGV browser view of H3K27me3 peak profiles across the entire X chromosome in wt *Tsix*-stop cells (blue) and KO cells (*Tsix-Kdm6a*^*ΔEΔE17*^) (red) at D0 and D15. The number of X chromosome peaks for each condition and time point are in parentheses. Green bars underneath represent genes along the X chromosome. The *Xist* gene is marked. **B** IGV browser view of H3K72me3 profiles and peaks at the *Xist* locus in wt *Tsix*-stop (blue) and KO *Tsix-Kdm6a*^*ΔEΔE17*^ cells (red) at D15. There is a slight increase in H3K27me3 levels in KO cells at D0, and a marked increase at D15. **C** Histogram of log_2_ fold change of *Xist* expression in wt (2 isolates: wt A, wt B) and two KO clones (*Tsix-Kdm6a*^*ΔEΔE17*^ and *Tsix-Kdm6a*^*ΔEΔE21*^) at D15 relative to D0. There is a markedly higher level of *Xist* induction in wt compared to KO clones. **D** IGV browser view of KDM6A profiles and peaks at D2, D4 and D7 of differentiation at the *Xist* locus in wt *Tsix*-stop cells. Merged data from two replicates of each time point are shown (see methods). KDM6A binding at the *Xist* gene promoter region and exon 1 is increased at D4 and lost at D7 following XCI. The scales of the profiles shown in **(A)** and **(B)** and **(D)** are indicated in the upper right corners

Next, we investigated changes in H3K27me3 profiles using Cut&Run to compare wt *Tsix-*stop cells to *Kdm6a* KO clone *Tsix-Kdm6a*^*ΔE/ΔE17*^. At D0 and D15, H3K27me3 levels (H3K27me3 peak numbers) appear similar along the whole X chromosome between wt and *Kdm6a* KO (Figure 3A), but a closer inspection revealed changes at the *Xist* locus. Initially (D0) H3K27me3 enrichment is only slightly higher across the *Xist* gene in *Kdm6a* KO ES cells, consistent with KDM6A’s role in resolving bivalent chromatin (Figure 3B). However, following XCI at D15, there is a marked increase in H3K27me3, which correlates with lower *Xist* expression that culminates at ~ 2.5 fold upregulation between D0 and D15 in KO cells, compared to ~ 6.0 fold in wt cells (Figure 3B, C). In addition to changes at *Xist*, H3K27me3 enrichment is also markedly higher at *Cdx4*, the *Tsix/Xist* locus, and the *Ftx* 5’ region in *Kdm6a* KO versus wt cells at D15 (Additional File 10: Figure S5A). Both *Cdx4* and *Ftx* show little expression change following *Kdm6a* KO, however, these genes are expressed at very low levels following differentiation (< 1TPM) and thus are not classified as DEGs (Additional File 10: Figures S5B–E). As a control, we analyzed a subset of known KDM6A target genes where we confirmed an increase in H3K27me3 levels associated with decreased expression in KO versus wt cells (Additional File 11: Figure S6A). Genes without significant expression change in KO cells did not show differences in H3K27me3, as expected (Additional File 11: Figure S6B). Consistent with our previous study, the imprinted *Dlk1/Meg3* locus showed an increase H3K27me3 levels in *Kdm6a* KO cells, which is specifically located along the imprinting control regions (ICR), and concordant with decreased gene expression (Additional File 11: Figure S6C; Additional File 3: Table S2) [14].

Chromatin analyses using Cut&Run with an antibody for KDM6A in wt *Tsix*-stop cells at D2, D4, and D7 show that KDM6A binding to the *Xist* promoter region increases at D4, when *Xist* expression levels continue to diverge between wt and *Kdm6a* KO cells, followed by a decrease at D7 (Figures 1C and 3D; Additional File 5: Figure S2A; Additional File 12: Table S6). A total of 582 KDM6A peaks mapped thoughout the entire genome in wt cells correspond to peaks of H3K27me3 enrichment, in contrast, in *Kdm6a* KO cells there is an increase in the number of H3K27me3 peaks that correspond to KDM6A peaks (870). This was observed both at D4 and D7, with a general decrease of KDM6A at D7 (Additional File 13: Figure S7A, B). Together, these results are consistent with KDM6A’s role in regulating H3K27me3 during cellular differentiation, such as in XCI, and with a genome-wide effect of *Kdm6a* KO on H3K27me3 levels (Additional File 13: Figure S7A, B). As expected, we observed KDM6A binding at promoters of known target genes with decreased expression in KO cells (Additional File 3: Table S3 and Additional File 13: Figure S7C) [14]. We next investigated the dynamics of KDM6A during early differentiation by qRT-PCR and protein blots, which showed a slight increase of both expression and protein levels during the early stages of differentiation followed by a decrease at D7 after XCI has occurred (Additional File 13: Figure S7D).

**Fig. 4:**
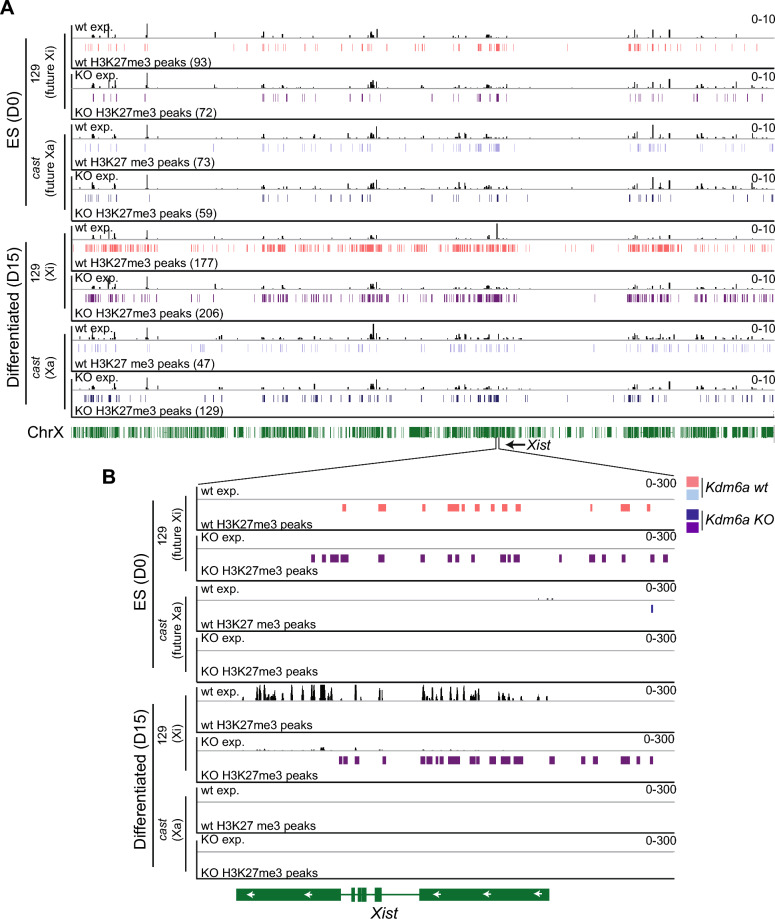
*Kdm6a* KO leads to increased H3K27me3 at *Xist* specifically on the Xi.** A** IGV browser views of gene expression (exp) and H3K27me3 peaks across the 129 and *cast* X chromosomes obtained by allelic analysis in *Tsix-*stop wt and KO cells (*Tsix-Kdm6a*^*ΔEΔE17*^) at D0 and D15. The 129 profile is in pink (wt) or purple (KO) and the *cast* in light blue (wt) or dark blue (KO). XCI is skewed in *Tsix*-stop cells so that the 129 X will become the Xi and the *cast* X the Xa. The number of peaks for each condition and time point are in parentheses. There is a marked increase in *Xist* expression from the Xi in wt cells but not in KO cells at D15. H3K27me3 levels increase following differentiation on the Xi in wt and *Kdm6a* KO cells. **B** Same analysis as in **(A)**, but zoomed in on the *Xist* gene. Allelic RNA-seq tracks of gene expression show very low expression from the 129 Xi allele in KO cells. A correlated Xi-specific increase in H3K27me3 enrichment at the *Xist* promoter is seen as an increase in peaks. No expression of *Xist* is observed from the *cast* allele, which is the Xa. The scales of the profiles are indicated in the upper right corners

Allelic effects of *Kdm6a* KO on H3K27me3 enrichment were investigated in *Tsix-*stop cells in which the Xi is from strain 129 due to skewed XCI [3]. At D0 few differences between alleles and between wt and *Kdm6a* KO are seen (Figure 4A). However, the future Xi (maternal 129 X) shows enrichment in H3K27me3 along *Xist*, which is not seen on the future Xa (paternal *cast* X) (Figure 4A, B). This is evident both in wt and KO cells at D0, and is consistent with previous studies demonstrating maternal-specific H3K27me3 enrichment at *Xist* [26]. At D15, wt cells show high overall H3K27me3 levels on the 129 Xi compared to the *cast* Xa, as expected due to the onset of XCI (Figure 4A; Additional File 14: Table S7). In *Kdm6a* KO cells there is both an increase and a decrease in H3K27me3, the latter being specifically associated with a number of genes that gained expression due to the lack of *Xist* expression and partial failure of XCI (Fig. 2B; Additional File 15: Figure S8). Overall, the gain in H3K27me3 on the 129 Xi in KO cells was modest. We used the wt sample as a background to call Xi peaks that appear in the KO sample. There were only 50 Xi-specific peaks gained in the KO cells that were absent in the wt, with the majority appearing either in gene bodies or at the Xist gene. As a result, only an additional 0.014% of the Xi was associated with an H3K27me3 peak. Interestingly, only the maternal allele of *Xist* shows persistence of higher levels of H3K27me3 following *Kdm6a* KO, which would explain decreased *Xist* expression and impaired XCI (Figures 4B; Additional File 14: Table S7). The observed genome-wide increase in H3K27me3 probably contributes to the large number of autosomal genes with decreased expression in *Kdm6a* KO cells versus wt (Figure 1B; Additional File 1: Figure S1B).

We conclude that prior to the onset of XCI, *Kdm6a* KO has a minimal effect on H3K27me3 levels at *Xist*. However, following differentiation persistence of high H3K27me3 levels would explain low levels of *Xist* expression. Although we cannot fully exclude indirect effects of *Kdm6a* KO on *Xis*t regulation, our data show association of KDM6A with the 5’ end of *Xist* during differentiation, suggesting that KDM6A may directly facilitate *Xist* expression during the female specific process of XCI.

## Discussion

Our study implicates KDM6A in the upregulation of *Xist* at the onset of XCI. We find that the female-biased histone demethylase helps *Xist* upregulation by binding to its promoter region and demethylating H3K27me3 during early mouse embryonic stem cell differentiation. In differentiated cells and adult tissues *Kdm6a* has higher expression in females compared to males due to escape from XCI, which may have evolved as a mechanism to regulate female-specific processes such as XCI [27]. Here, we show that KDM6A facilitates upregulation of *Xist* during early stages of XCI in hybrid mouse ES cell models with skewed or near-random XCI. *Kdm6a* KO leads to increased H3K27me3 levels on the maternal allele of *Xist* whose expression and ability to coat the Xi decrease following differentiation. In turn, this failure of XCI results in aberrant expression of genes from the Xi. Increased X-linked gene expression due to impaired XCI due to KDM6A deficiency may contribute to phenotypes in a variety of tissues such as those observed in Kabuki and Turner syndromes in human [28, 29].

The ES cell line in which we followed *Xist* expression and silencing of X-linked genes has skewed XCI of the maternal 129 allele due to a stop mutation at *Tsix.* It could be argued that this mutation may affect our observations. However, we found that an independent *Kdm6a* KO line without a *Tsix* mutation also shows impaired *Xist* expression and X silencing. Our findings are consistent with previous studies that have shown that maternal imprinting of *Xist* is maintained by maternal-specific H3K27me3 enrichment at *Xist* in blastocysts and that demethylation is critical for initiation of *Xist* expression and XCI [26]. Indeed, injection of *Kdm6b,* an autosomal gene that encodes another H3K27me3 demethylase, in preimplantation embryos results in maternal allele-specific loss of H3K27me3 and induction of *Xist* expression [26]. Therefore, removal of the repressive chromatin mark appears necessary to initiate random XCI. However, the question of how the maternal imprint at the *Xist* locus is maintained in the blastocyst remains. One possibility is that the increased levels of H3K27me3 on the maternal allele in wt *Tsix*-stop ES cells we observe is due to the influence of Xce. Here, we used hybrid mESCs with different X chromosome controlling element (*Xce*) [30]. However, the 129 X chromosome (maternal in this model) is more likely to be inactivated compared to the *cast* X chromosome (paternal is this model) [30]. Therefore, if Xce differences played a major role in biased H3K27me3 levels here, we would expect increased H3K27me3 on the *cast* allele instead. Another possibility is that KDM6A may play a role in the erasure of the imprint at *Xist* in mESCs with KO resulting in re-establishment of the imprint, but experiments in mESCs derived from a reciprocal cross would be needed to confirm this. In addition, we have previously demonstrated that KDM6A regulates expression of maternally expressed X-linked *Rhox* genes in a female-specific manner via removal of H3K27me3, and preferentially regulates maternal alleles of autosomal genes [14, 31]. Transcriptional regulation by KDM6A can occur through histone demethylation-dependent and -independent mechanisms [20]. In contrast, the protein encoded by the Y-linked homolog of *Kdm6a*, UTY, lacks detectable demethylase activity, suggesting that female-biased expression of *Kdm6a* may have evolved female-specific functions requiring H3K27me3 demethylation [15, 20]. Consistent with this, homozygous knockout (KO) of *Kdm6a* is lethal in female mice, while a small number of runted males with hemizygous deletion survive, suggesting that UTY partially compensates for loss of KDM6A through demethylation-independent mechanisms [32].

XCI is necessary for loss of pluripotency and normal differentiation, a mechanism that depends on expression of specific genes [4, 33, 34]. We find that germ layer marker genes have lower expression of *Kdm6a* in KO cells than in wt, which is consistent with previous analyses demonstrating that KDM6A impairs differentiation. Indeed, *KDM6A* mutations are associated with increased “stemness” in cancer and *Kdm6a* KO impairs mesoderm development *in vivo* largely through histone demethylase-independent mechanisms [20]. However, recent studies of *Kdm6a* KO models indicate that depletion of the enzyme does not block differentiation, and may even facilitate differentiation to neuroectoderm lineages [35, 36]. Our analysis in an additional cell line E8 with *Kdm6a* KO revealed no significant trend of downregulation of germ layer genes at time points prior and during XCI. Indeed, we only observed effects on germ layer marker genes at D15 after XCI has occurred, and our KDM6A chromatin binding analyses suggest a direct effect of KDM6A on *Xist* expression during early XCI rather than an indirect effect via a block on differentiation. In support, allelic single cell RNA-seq analyses in differentiating hybrid mouse ES cells show that XCI is complete prior to lineage specification, suggesting that the effects of *Kdm6a* KO on *Xist* expression we observe occur before lineage determination [4]. Thus, our results indicate that impaired XCI is due to failure of *Xist* upregulation in *Kdm6a* KO cells leading to abnormally high dosage of X-linked genes, which may then cause a block in differentiation at later time points [37]. However, increased expression of *Klf4* and decreased expression of lineage-specific genes following *Kdm6a* KO suggests a potential delay in differentiation, therefore we cannot fully decouple the potential effects of *Kdm6a* KO on differentiation from the effects on regulation of *Xist* expression.

In undifferentiated ES cells the *Xist* promoter is maintained in a bivalent state, which is probably targeted for resolution by KDM6A via removal of H3K27me3 to facilitate expression during initiation of XCI [13, 38–40]. Consistent with this hypothesis, H3K27me3 levels show an increase in KO cells. A number of repressive epigenetic events occur following coating of the Xi by *Xist* including deposition of H3K27me3 by PRC2 [2]. One interesting possibility is that KDM6A, in addition to resolving bivalent chromatin for activation, also serves to help protect the *Xist* locus from being silenced during the XCI process by actively removing H3K27me3 during the process of XCI. However, additional studies are needed to fully address that possibility. KDM5C, which is also encoded by an escape gene, has recently been implicated in facilitating *Xist* expression by converting H3K4me3 to H3K4me1 at the *Xist* 5’ end [7]. Interestingly, KDM5C appears to be enriched at a similar location on the *Xist* gene as KDM6A, suggesting potential cooperation between the two proteins, each encoded by an escapee, that may help form a permissive chromatin environment for *Xist* expression. This previous study also points to a threshold of higher expression of escapees in females necessary for XCI. Consistent with this, our heterozygous depletion of *Kdm6a* leads to reduced *Xist* upregulation similar to our homozygous KO, albeit at a later time point. KDM6A may also regulate the expression of other regulatory elements located in the X inactivation center (XIC), such as *Ftx* and *Jpx,* two escape genes with roles in *Xist* regulation [41]*.* Transcription of the lncRNA *Ftx* promotes *Xist* activation during initiation of XCI [42]. While *Ftx* has very low expression in *Tsix-*stop ES cells (< 1TPM), we did observe an increase of H3K27me3 levels at the *Ftx* promoter and a slight decrease in *Ftx* expression following *Kdm6a* KO, which could contribute to a decrease in *Xist*. *Tsix* is another important regulator of *Xist*. However, the consistently lower expression of *Tsix* we observed during early differentiation of *Kdm6a* KO cells compared to wt, indicates that the decrease in *Xist* expression is independent of *Tsix-*induced repression [43].

The polycomb complexes PRC1/2 that mediate methylation of H3K27 have been implicated in the dynamics of XCI and maternally imprinted genes [26, 44, 45]. Indeed, the absence of polycomb group proteins due to lack of *Xist*-mediated recruitment leads to a relaxation of transcriptional silencing [46]. Consistent with these results, we observe a significant upregulation of gene expression from the Xi following differentiation in *Kdm6a* KO cells. A trend of increased expression from the Xi was seen for all temporally defined clusters of silenced genes, as well as genes in the escape category. Genes that escape XCI rarely achieve a level of Xi expression equal to that from the Xa due the repressive chromatin environment of the Xi [28]. Thus, the effects of *Kdm6a* KO on genes that escape may be due to an overall relaxation of the Xi heterochromatin. Our Xi-specific analysis of H3K27me3 indicates that KDM6A’s main action in XCI is on upregulation of Xist itself, and not through regulation of individual X-linked genes. Indeed, increased gene expression from the Xi was not accompanied by a clear reduction of H3K27me3 peaks on the Xi in differentiated *Kdm6a* KO cells compared to wt. It is possible that depletion of Xist, together with the absence in histone demethylation on the Xi due to KDM6A KO results in relatively balanced Xi H3K27me3 levels relative to wt.

Here, we illustrate a novel female-specific role for the histone demethylase KDM6A in the threshold dependent regulation of *Xist* expression for the initiation of XCI through its histone demethylation-dependent function. A recent study also points to a role for KDM6A in regulating *XIST* expression in human disease [47]. Therefore, extending our studies to human models will help determine the role of KDM6A in regulating X-linked gene expression in cases of sex chromosome aneuploidy as well as Kabuki syndrome and in cancer, where it is frequently mutated.

### Perspectives and significance

This study identifies KDM6A as a female-biased novel member of the *Xist* regulome by demonstrating that KDM6A helps increase *Xist* expression by removing a repressive histone mark H3K27me3 at the *Xist* promoter region. The female-biased expression of *Kdm6a* may have evolved as a mechanism to enhance female-specific processes such as X inactivation. Interestingly, *Kdm5c*, another gene that also escapes X inactivation and encodes another demethylase critical for enhancer and promoter activity, has also been implicated in facilitating *Xist* expression. Thus, KDM6A and KDM5C may cooperate in enhancing *Xist* expression at a critical time during early development to ensure silencing of the X chromosome in females. KDM6A appears to have both histone demethylase-dependent and -independent activities, which are not well understood. Future studies should include additional epigenetic analyses of histones and of other chromatin components, together with analyses of the 3D conformation of chromatin, which may help ascertain the complex network of gene regulation by KDM6A. Our findings may have implications for the understanding of sex differences in disease, including sex-biased immune disorders where X inactivation is often incomplete. Finally, extending our studies to human stem cell models will help understand the role of KDM6A in disease.

## Conclusions

Here, we demonstrate a novel role for the female-biased H3K27me3 demethylase KDM6A in the regulation of the female-specific process of X chromosome inactivation. We show that depletion of KDM6A prevents *Xist* upregulation during early differentiation, resulting in reduced efficacy of X chromosome silencing. Thus, escape from X inactivation may have evolved in part to perform essential roles in female-specific processes such as X inactivation.

## Methods

### Cell culture and differentiation

The female F1 hybrid mouse ES line *Tsix-*stop was derived from a cross between 129 and *cast* followed by the insertion of a transcriptional stop in the *Tsix* gene on the 129 X chromosome [18]. Differentiation of *Tsix-*stop ES cells results in skewed XCI where the Xi is always the 129 (maternal) X chromosome. In contrast, differentiation of the female F1 hybrid mouse ES line E8, which is derived from a cross between C57BL/6J (B6) and *cast*, results in largely random XCI. ES cells (*Tsix*-stop, E8) were maintained in the presence of 1000 U/ml leukemia inhibitory factor (LIF) (Millipore) on a monolayer of chemically inactivated mouse embryonic fibroblasts (MEFs), in high glucose DMEM media supplemented with 15% fetal bovine serum (FBS), 1% non-essential amino acids, 10 mg/ml APS, 0.1mM 2mercaptoethanol and 25 mM l-glutamine, in a humidified incubator at 37 ºC and 5% CO_2_. Just prior to use, ES cells were enriched by incubation on 0.1% gelatin-coated dishes for 1h to allow MEFs to attach, followed by transfer to fresh gelatin-coated plates for overnight culture. Following expansion, ES cells were split once (1:10) to further reduce potential MEF contamination. For embryoid body (EB) formation, 4x10^6^ wt control and *Kdm6a* KO cells were cultured on non-adherent bacterial culture dishes without LIF for 7 days. Cells were collected at D0, D2, D4, and D7 of differentiation. Cells were then plated onto gelatin-coated cell culture plates and maintained for another 8 days prior to collection at D15.

### *Kdm6a* editing using CRISPR/Cas9

We chose a dual sgRNA approach because of the capacity to create large deletions and reduced chance of off-target effects [48]. Editing of *Kdm6a* in *Tsix-*stop ES cells was done by constructing CRISPR plasmids containing sgRNAs that target exons 2 and 4 to delete ~ 45kb, as done previously [14, 49]. ES cells were co-transfected with the CRISPR/Cas9 constructs and a plasmid carrying puromycin resistance using UltraCruz® transfection reagent at a 3:1 CRISPR to pPGKpuro ratio in media with no antibiotics. Two days later, cells were selected in ES media containing 1 μg/ml puromycin for 48–72 h, followed by recovery in media with antibiotic. Cells were then cloned into 96-well plates using serial dilutions. Clones were expanded and screened for deletions using PCR and Sanger sequencing to confirm the predicted ~ 45 kb deletion, which was found in three clones including a heterozygous deletion clone (*Tsix-Kdm6a*^*wt/ΔE4*^) and two homozygous deletion clones (*Tsix-Kdm6a*^*ΔE/ΔE17*^ and *Tsix-Kdm6a*^*ΔE/ΔE21*^) (Additional File 1: Figure S1A; Additional File 2: Table S1). Additionally, we confirmed that neither *Tsix-*cln1 nor *Tsix-*cln2 CRISPR controls were edited (Additional File 1: Figure S1A). Editing of *Kdm6a* in E8 ES cells was done by targeting the *Kdm6a* promoter region using sgRNAs designed using the CHOPCHOP v2 online tool [50]. PCR amplification and Sanger sequencing confirmed the predicted ~5 kb homozygous promoter deletion in one clone (E8-*Kdm6a*^*ΔP/ΔP13*^) (Additional File 1: Figure S1A; Additional File 2: Table S1). Plasmid preparations were made using either the Qiagen Mini-prep or Maxi-prep kits according to the manufacturer protocol. Plasmid DNA and RNA lysates were submitted to Eurofin Genomics for Sanger sequencing (http://www.operon.com).

### DNA, RNA and protein isolation and analyses

RNA and DNA lysates were prepared using Qiagen RNeasy mini kit and the Qiagen DNeasy blood and tissue kit, respectively. cDNA synthesis was done with the GoScript reverse transcription kit (Promega). Relative transcript levels were determined using SYBR Green PCR master mix on an ABI 7900HT machine. qRT-PCR assays were conducted in triplicates and normalized to *Actinβ* prior to analysis using the comparative CT method. For protein analyses, western blots were performed using a monoclonal antibodies directed against KDM6A (Cell Signaling #33510) at 1:1000 and GAPDH (GeneTex #GTX627408) at 1:10000 dilution in 5% BSA according to standard methods. 30ug of total protein was added to each lane and visualized with Clarity ECL (Bio-Rad #1705060). Band densities were calculated using Imagej (https://imagej.net/ij/).

### DNA-FISH and RNA-FISH

To verify the presence of two X chromosomes in differentiated wt and CRISPR-treated cells, DNA-FISH was done on nuclei fixed in methanol:acetic acid (3:1 volume) using BAC probes (RP23-299L1 for minisatellite repeats or RP24-322N20 for *Firre*) [51]. Probes were labeled with SpectrumGreen (Vysis #02N32-050) using a nick-translation reagent kit according to the manufacturer’s protocol (Abbott Molecular Inc.). Probes in hybridization buffer were denatured at 70 °C for 5 min and hybridized overnight at 37 °C to slides that were also denatured at 70 °C for 5 min prior to hybridization. On the next day, slides were washed in SSC containing NP40 at 73 °C, followed by washes at room temperature. Counterstaining was done with Hoechst 33342 (2 µg/ml) in phosphate buffered saline (PBS) and slides were mounted in anti-fade solution.

For *Xist* RNA-FISH cells were plated on chamber slides at D7 of differentiation and allowed to differentiate until D15 when cells were processed directly on the chamber slides as described [52]. Briefly, cells were washed in PBS and permeabilized in CSK buffer with 0.05% Triton. Following fixation in 4% paraformaldehyde, cells were washed in 70% ethanol and dehydrated for 3 min each in 70%, 85%, and 100% ethanol. Dehydrated slides were incubated with a denatured probe for *Xist* RNA overnight in 50% formamide/50% 2xSSC at 42  °C in a humidified chamber [52]. On the following day, slides were washed three times in 50% formamide/50% 2xSSC at 42 °C for 10 min followed by one wash in 2xSSC at 42 °C for 5 min. Counterstaining was done with 33342 Hoechst in 2xSSC. A minimum of 130 nuclei were scored for each condition to quantify the number of nuclei with either an *Xist* cloud, a pinpoint *Xist* signal, or no *Xist* signal.

### RNA-seq and allelic gene expression analysis

RNA-seq indexed libraries were prepared using Illumina TruSeq RNA sample preparation kit V2. For *Tsix-*stop cells, libraries were prepared from two biological replicates from wt controls, one CRISPR control (cln1) and *Tsix*-*Kdm6a*^*EΔ/EΔ17*^ and *Tsix*-*Kdm6a*^*EΔ/EΔ21*^ KO clones at D0 and D15. For E8 cells, libraries were prepared from one wt control and one E8-*Kdm6a*^*ΔP/ΔP13*^ clone at D0, D2 and D4. Sequencing on a NextSeq sequencer yielded 75bp single-end reads. Diploid gene expression was estimated using Tophat/v2.0.14 [53] with default parameters and gene-level expression was normalized using TPM (transcripts per kilobase of exon length per million mapped reads). Differentially expressed genes were determined using DESeq2 analysis [54] with a false discovery rate (FDR) threshold of 0.1 and a fold-change cutoff of 2. PCA plots and heatmaps were generated with iDEP.95 using raw read counts as the input. Only genes with a minimum expression value of 2.5 CPM in at least two libraries were included (http://bioinformatics.sdstate.edu/idep95/).

To estimate allele-specific gene expression in mouse hybrid cells, the pseudo-diploid genome of 129x*cast* was generated using GATK-fastagenerator subcommand [55], taking the mm10 reference genome fasta file and the corresponding VCF file as the input. The VCF file was downloaded from the Sanger mouse genomes project [56], with indels and low-quality SNPs filtered out. RNA-seq reads were mapped using bowtie2 [57] to the diploid pseudo-129 and pseudo-*cast* genomes and transcriptomes. Only those reads that mapped uniquely and with a high-quality mapping score (MAPQ ≥ 30) were kept for further analyses. After filtering, reads were segregated into three categories: (1) 129-SNP reads containing only 129-specific SNPs; (2) *cast*-SNP reads containing only *cast*-specific SNPs; (3) allele-uncertain reads, that is, reads that do not contain valid SNPs. Allelic mapping metrics are shown in Additional File 16: Table S8.

### Epigenetic analyses by CUT&RUN

Cut&Run for KDM6A was done in wt cells using an antibody against KDM6A (Cell signaling #33510) according to the manufacturer’s protocol (EpiCypher) except with mild fixation (0.01% formaldehyde for 1 min). Cut&Run for H3K27me3 was performed on clone *Tsix-Kdm6a*^*ΔE/ΔE17*^ and wt cells using an antibody against H3K27me3 (Millipore) following a published protocol [58]. Libraries were constructed using TruSeq DNA sample preparation kit (Illumina) following the manufacturer protocol with minor changes including two 1.1x bead purifications during the final selection step.

Libraries were sequenced on a NextSeq sequencer to generate 75bp paired-end reads. In the following text, we refer to paired-end reads simply as “reads”. Demultiplexed fastq files were mapped to NCBi v38/mm10 mouse genome using bowtie2 [57] with the following parameters:

––end–to–end ––very–sensitive ––no–mixed ––no–discordant ––phred33 –I 10 –X 700.

Only paired reads that were mapped to the same chromosome with a fragment length of less than 1 kb were kept for further analyses. Read alignment BAM files were converted to bigWig format for IGV visualization using Bedtools [59]. Read counts were normalized using RPKM; and IgG control was subtracted from the knockout sample using the bamcompare subcommand in deeptools [60]. BAM files of KDM6A Cut&Run replicates for D2, D4, and D7 were merged using SAMtools to facilitate peak calling. The read counts in the BigWig files were normalized based on RPKM. Peak calling for KDM6A and H3K27me3 samples was performed using SEACR [61] on bedGraph files, applying the “norm” option.

$$RPKM= \frac{Number\;of\;reads\;mapped\;to\;each\;base}{\frac{total\;mapped\;reads\;in\;the\;sample}{100000000}}$$

For allelic-analysis, the pseudo-diploid genome of 129 × *cast* was generated using GATK-fastagenerator subcommand [55], taking the mm10 reference genome fasta file and the corresponding VCF file as the input. The VCF file was downloaded from the Sanger mouse genomes project [56], with indels and low-quality SNPs filtered out. Reads were mapped to the diploid pseudo-129 and pseudo-*cast* genomes using bowtie2 [57] with the following parameters:

––end–to–end ––very–sensitive ––no-mixed ––no–discordant –phred33 –I 10 –X 700.

Reads were further processed through a read tagging step, where read alignments of both pseudo-129 and pseudo-*cast* genomes were compared to identify the allele origins of the reads. Reads overlapping SNPs were assigned to G1 (129 genome), G2 (*cast*), or CF (conflicting) based on the sequenced base information at SNP sites, while reads do not overlap with any SNPs were assigned to UA (Unassigned). The G1 and G2 tagged reads were separated into two BAM files, which were later used to generate allele-specific bigWig files for IGV visualization [62]. An allelic mapping summary can be found in Additional File 17: Table S9.

### Analysis of overlapping KDM6A and H3K27me3 peaks

We called peaks for all samples using SEACR as described, filtering out H3K27me3 peaks with a signal lower than 10 for the D15 samples. Due to lower coverage, peaks with a signal lower than 5 for the KDM6A sample were excluded. Transcription start sites (TSS) were defined as regions extending 5 kb upstream and downstream from the transcript start site. TSS regions containing peaks were extracted for each sample. These sample-specific TSS regions were used to identify both sample-specific and shared peaks across two or three samples for Venn diagram plotting. Only the number of genes rather than transcripts was considered.

## Supplementary Information

**Additional file 1: Supplemental figure S1. CRISPR-Cas9 strategy and characteristics of Kdm6a KO cells. (A)** Top: Schematic shows the location of the exonic deletion (*Kdm6a*^*ΔE*^) that removed exons 2–4 of *Kdm6a* in female *Tsix-*stop ES cells and the promoter targeted deletion (*Kdm6a*^*ΔP*^) made in female E8 ES cells. Exons are shown as vertical bars. The location of the PCR and RT-PCR primers (color-coded arrows) used to confirm each deletion is indicated. Below left: Images of gels after electrophoresis of PCR products using different sets of primers to confirm *Kdm6a* homozygous and heterozygous KO (- no deletion; + deletion positive). *Actinβ* was run as a control. Below right: Partial *Kdm6a* sequence obtained by Sanger sequencing as verification of deletions in *Tsix*-stop and E8 homozygous *Kdm6a* KO clones. Arrows point to the location of non-homologous end-joining and colored arrows correspond to those on the schematic. **(B)** Heat maps of gene expression differences between *Tsix-*stop wt (2 replicates, wt a and wt b) and KO clones (*Tsix-Kdm6a*^*ΔEΔE17*^ and *Tsix-Kdm6a*^*ΔEΔE21*^) at D0 and D15. Consistent with PCA clustering, more DEGs were identified at D15 than D0. Heat maps were generated using iDEP.95. **(C)** Scatter plots of expression of genes involved in germ layer differentiation obtained by RNA-seq. Tsix-*stop* wt and KO cells after differentiation (D15) show higher expression of germ layer-associated genes in wt versus KO cells (*Tsix-Kdm6a*^*ΔEΔE17*^ and *Tsix-Kdm6a*^*ΔEΔE21*^*)*. Log_2_ average TPM values are from two wt replicates and two KO clones. E8-*Kdm6a* KO cells show no trend of significant difference in expressed germ layer genes at D2 or D4 of differentiation. P-values are calculated using 1-way ANOVA test.

**Additional file 2.**

**Additional file 3.**

**Additional file 4.**

**Additional file 5:Supplemental figure S2. Confirmation of Xist expression changes in Kdm6a KO cells. (A)** qRT-PCR of *Xist* expression normalized to *Actinβ* during ES differentiation in three *Tsix*-stop wt replicates, the two *Kdm6a*^*ΔEΔE*^ KO clones, and one *Kdm6a*^*ΔwtΔE*^clone. Shown is expression over time for each individual sample. **(B)** Histogram of qRT-PCR for *Xist* expression during differentiation in *Tsix-*stop wt and CRISPR controls (Additional File 1: Table S1). Expression is normalized to *Actinβ*. Expression at D4 is relative to D0 for each cell line. As expected, *Xist *is upregulated upon differentiation in wt (wta and wtb replicates) and CRISPR-control clones (cln1 and cln2).** (C)** Histogram of log_2_of average TPM values for pluripotency genes and genes known to play a role in *Xist* repression. Average TPM values are from two *Tsix-*stop wt and two *Kdm6a* KO clones (*Tsix-Kdm6a*^*ΔEΔE17*^ and *Tsix-Kdm6a*^*ΔEΔE21*^). Only *Klf4* is called as differentially expressed between wt and KO by DESeq2 (***p<0.002). **(D, E)** Histograms of qRT-PCR of *Tsix *expression in **(D)** *Tsix*-stop wt and *Kdm6a*^*ΔEΔE*^ KO clones, and **(E)** E8 wt and KO clone E8-*Kdm6a*^*ΔP/ΔP13*^ at D2 and D4. Expression is normalized to *Actinβ* and relative to wt.** (F)** Histogram of average TPM values for *Xist* in *Tsix-*stop wt, a CRISPR-control clone (cln1), and two KO clones (*Tsix-Kdm6a*^*ΔEΔE17*^, *Tsix-Kdm6a*^*ΔEΔE21*^*)* at D0, confirming that *Xist* expression is very low in ES cells cultured with serum.

**Additional file 6 Supplemental figure S3. Two X chromosomes are present in wt and KO Tsix-stop cells. (A, B)** DNA-FISH using probe*s* specific for the X-linked gene *Firre* or an X-linked mini-satellite repeat region (*Dxz4*) labelled in green in *Tsix*-stop wt **(A)** and CRISPR-control (cln1) **(B)**. Examples of nuclei with two green signals representing the two X chromosomes are shown along with histograms of the number of signals in nuclei scored. n indicates the number of nuclei scored. Nuclei are counterstained with Hoechst 33342. **(C, D)** Same analysis in as in **(A, B)**, but in the *Tsix*-stop *Kdm6a* KO cell clones (*Tsix-Kdm6a*^*ΔEΔE17*^ and* Tsix-Kdm6a*^*ΔEΔE21*^).

**Additional file 7 Supplemental Figure S4. Confirmation of X-linked gene expression changes in E8-Kdm6a**^***ΔPΔP13***^** KO cells. (A) **Allelic X-linked gene expression ratios (BL6:*cast*) in E8 wt and KO cells (*E8-Kdm6a*^*ΔPΔP13*^) at D0, D2, and D4 of differentiation. Ratios between expressed X-linked genes (>1TPM) are shown. X expression ratios near 0.5 reflect the largely random nature of XCI in these cells. **(B)** Plots of expression changes from the BL6 and cast X chromosomes in E8 wt and KO cells (*E8-Kdm6a*^*ΔPΔP13*^) at D4. The XIC is highlighted. Log2 TPM fold change (KO/wt) along the X chromosomes is shown. Genes with a decrease of expression in KO versus wt are in blue, and with an increase in red. **(D)** Venn diagram of the number of X-linked genes with upregulation following *Kdm6a* KO. The majority of genes are upregulated from the BL6 and *cast* X chromosomes.** (D)** Plots of expression changes (based on log2 TPM) for genes with increased expression from each X chromosome (BL6 purple and cast blue) between E8 wt and E8-*Kdm6a*^*ΔPΔP13*^ KO cells at D4. The X-axis includes 254 X-linked genes. There is a slightly greater increase in expression from the BL6 X chromosome (purple).

**Additional file 8.**

**Additional file 9.**

**Additional file 10 Supplemental figure S5.** **H3K27me3 profiles along the XIC in wt and Kdm6a KO cells. (A)** IGV browser view of profiles and peaks of H3K27me3 enrichment at the XIC (chrX: 103126305-103891745) show increased H3K27me3 at *Cdx4,* *Xist,* and *Ftx* in differentiated *Kdm6a* KO cells. *Tsix-*stop wt (blue) and KO clone *Tsix-Kdm6a*^*ΔEΔE17*^ (red). **(B, C)** Histograms of expression (TPM) of *Cdx4* **(B)** and *Ftx* **(C)** in D0 *Tsix-*stop wt (blue) and *Kdm6a* KO clones (red). Both wt and KO ES cells show low expression of *Cdx4* and *Ftx* (~1.5TPM or less). **(D, E)** Same analysis as in **(B)** and **(C)** but for D15. Both wt and KO ES cells show low expression of *Cdx4* and *Ftx* (~1TPM or less).

**Additional file 11 Supplemental figure S6. Epigenetic and expression changes at known KDM6A target genes. (A)** IGV browser views of profiles of H3K27me3 enrichment and peaks at known KDM6A target genes in differentiated *Tsix-*stop wt cells (blue) and KO clones (*Tsix-Kdm6a*^*ΔEΔE17*^) (red). Peaks are represented by colored bars below the signal profiles. Below are RNA-seq expression profiles (black) in wt and KO cells. Histograms show TPM expression values for *T* and *Pitx2* in two differentiated wt clones and KO clones. For *Wnt3* and *Gata4*, both changes in expression at D0-7 obtained by qRT-PCR during differentiation and histograms of TPM values following differentiation are shown (*p<0.05). KO represents an average for the *Tsix-Kdm6a*^*ΔEΔE17*^ and *Tsix-Kdm6a*^*ΔEΔE21*^ clones*.* Values are normalized to *Actinβ.* **(B)** Same analysis as in **(A)**, but for *Nsdhl* and *Araf*, two genes that do not show significant downregulation following *Kdm6a* KO.** (C)** Same analysis as in **(A)**, but at the *Dlk1*/*Meg3* imprinted locus at D15 where H3K27me3 markedly increases at the promoter of *Meg3* in KO cells, including at the imprinting control region ICR (2) and the intergenic ICR (1). The scales of the profiles shown in **(A)**, **(B)** and **(C)** are indicated in the upper right corners.

**Additional file 12.**

**Additional file 13 Supplemental figure S7. Characterization of KDM6A binding, expression and protein levels during differentiation of Tsix-stop cells. (A) **Venn diagram comparing number of peaks of KDM6A in wt *Tsix*-stop cells at D4 of differentiation to H3K27me3 peaks in wt and *Kdm6a* KO cells at D15 of differentiation.** (B) **Same analysis as in **(A)** but using KDM6A binding at D7 for comparisons.** (C) **KDM6A enrichment profiles at *Foxr2* and *Igsf9*, two genes known to be regulated by KDM6A. D4 signal and peaks in wt *Tsix*-stop cells are shown. Scales are shown in the upper right corner. **(D)** Top, protein blots of KDM6A in wt *Tsix*-stop (top) during differentiation. Adjusted density was calculated by normalizing levels to the GAPDH loading control using ImageJ (middle). Bottom, histograms of qRT-PCR of *Kdm6a* expression in two wt *Tsix*-stop replicates during differentiation. Expression was normalized using *Actinβ* and expression relative to D0 is shown.

**Additional file 14.**

**Additional file 15: Supplemental figure S8.** **Allelic H3K27me3 enrichment at X-linked genes with Xi-specific expression increases in Kdm6a KO Tsix-stop cells.** IGV browser views of Xi H3K27me3 signal and peaks at a subset of genes with increased expression from the Xi in *Kdm6a* KO cells. Wt cells are in pink and KO cells are in purple. Some genes with Xi-specific increases in expression following differentiation (D15) show lack of H3K27me3 near their TSS, due to failure of XCI and recruitment of PRC complexes.

**Additional file 16.**

**Additional file 17.**

## Data Availability

All sequencing data that support findings in this study have been deposited in the NCBI GEO database under the accession numbers GSE279278 and GSE279279. All other data and the scripts used for the analyses that support the findings of this study are available from the corresponding authors upon reasonable request. Oligonucleotide sequences used in this study are available in Additional File 2: Table S1.
