# Supplementary figures and images for: KDM6A facilitates Xist upregulation at the onset of X inactivation
Source: Biol Sex Differ. 2025 Jan 3;16:1. doi: 10.1186/s13293-024-00683-3 (PMC11699772; doi:10.1186/s13293-024-00683-3)

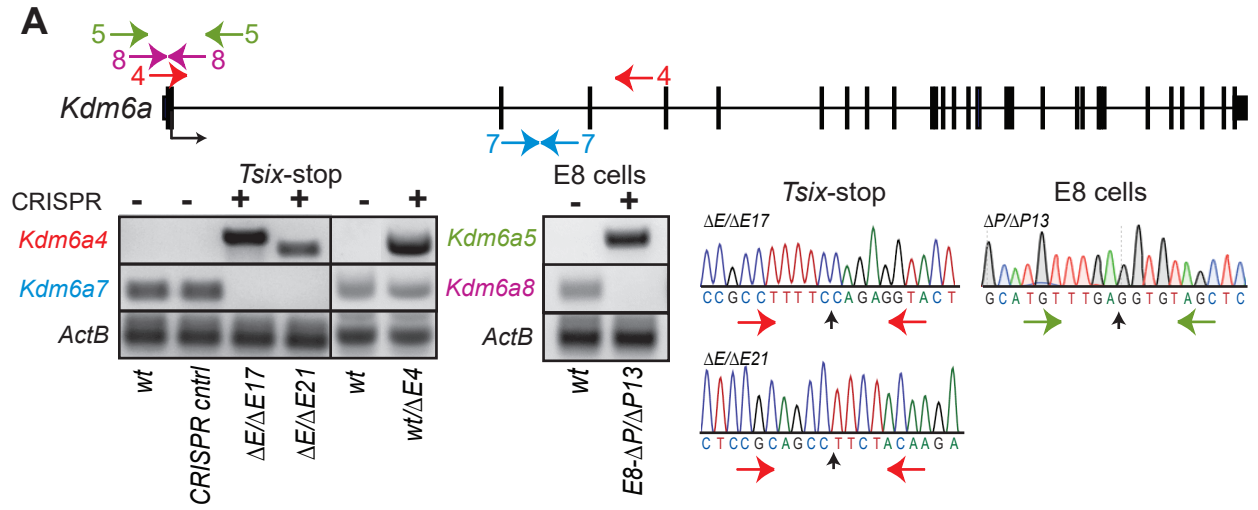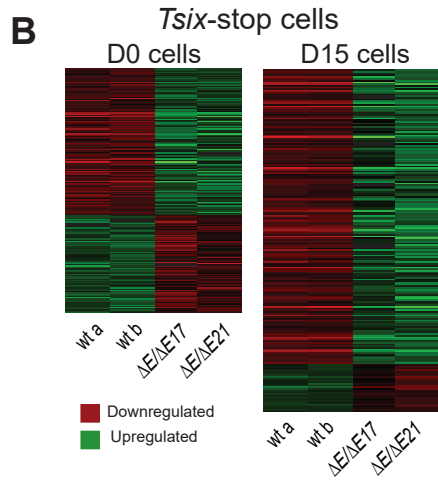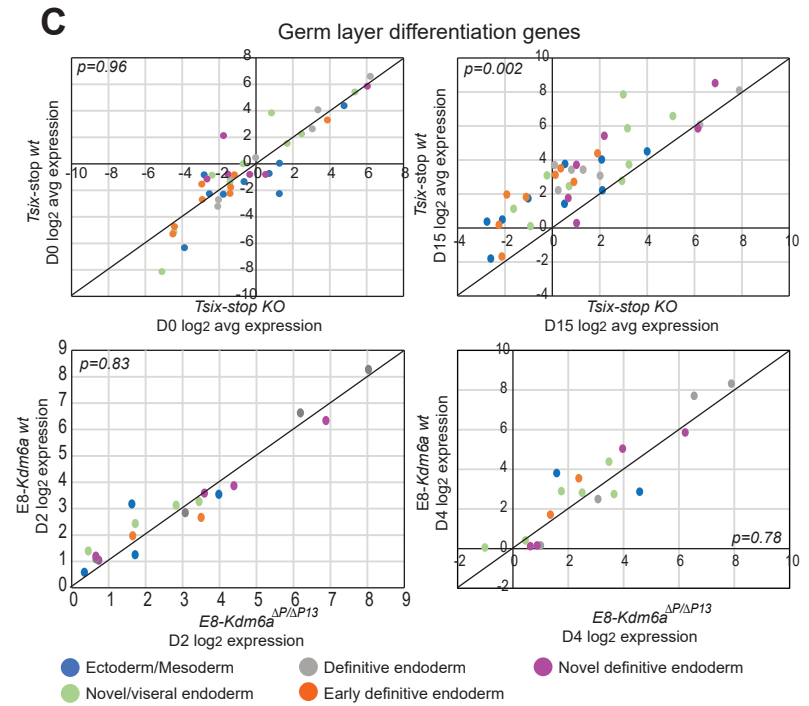

Supplement: Supplementary file 1 — Additional file 1: Supplemental figure S1. CRISPR-Cas9 strategy and characteristics of Kdm6a KO cells. (A) Top: Schematic shows the location of the exonic deletion (Kdm6aΔE) that removed exons 2–4 of Kdm6a in female Tsix-stop ES cells and the promoter targeted deletion (Kdm6aΔP) made in female E8 ES cells. Exons are shown as vertical bars. The location of the PCR and RT-PCR primers (color-coded arrows) used to confirm each deletion is indicated. Below left: Images of gels after electrophoresis of PCR products using different sets of primers to confirm Kdm6a homozygous and heterozygous KO (- no deletion; + deletion positive). Actinβ was run as a control. Below right: Partial Kdm6a sequence obtained by Sanger sequencing as verification of deletions in Tsix-stop and E8 homozygous Kdm6a KO clones. Arrows point to the location of non-homologous end-joining and colored arrows correspond to those on the schematic. (B) Heat maps of gene expression differences between Tsix-stop wt (2 replicates, wt a and wt b) and KO clones (Tsix-Kdm6aΔEΔE17 and Tsix-Kdm6aΔEΔE21) at D0 and D15. Consistent with PCA clustering, more DEGs were identified at D15 than D0. Heat maps were generated using iDEP.95. (C) Scatter plots of expression of genes involved in germ layer differentiation obtained by RNA-seq. Tsix-stop wt and KO cells after differentiation (D15) show higher expression of germ layer-associated genes in wt versus KO cells (Tsix-Kdm6aΔEΔE17 and Tsix-Kdm6aΔEΔE21). Log2 average TPM values are from two wt replicates and two KO clones. E8-Kdm6a KO cells show no trend of significant difference in expressed germ layer genes at D2 or D4 of differentiation. P-values are calculated using 1-way ANOVA test. [file 13293_2024_683_MOESM1_ESM.pdf]

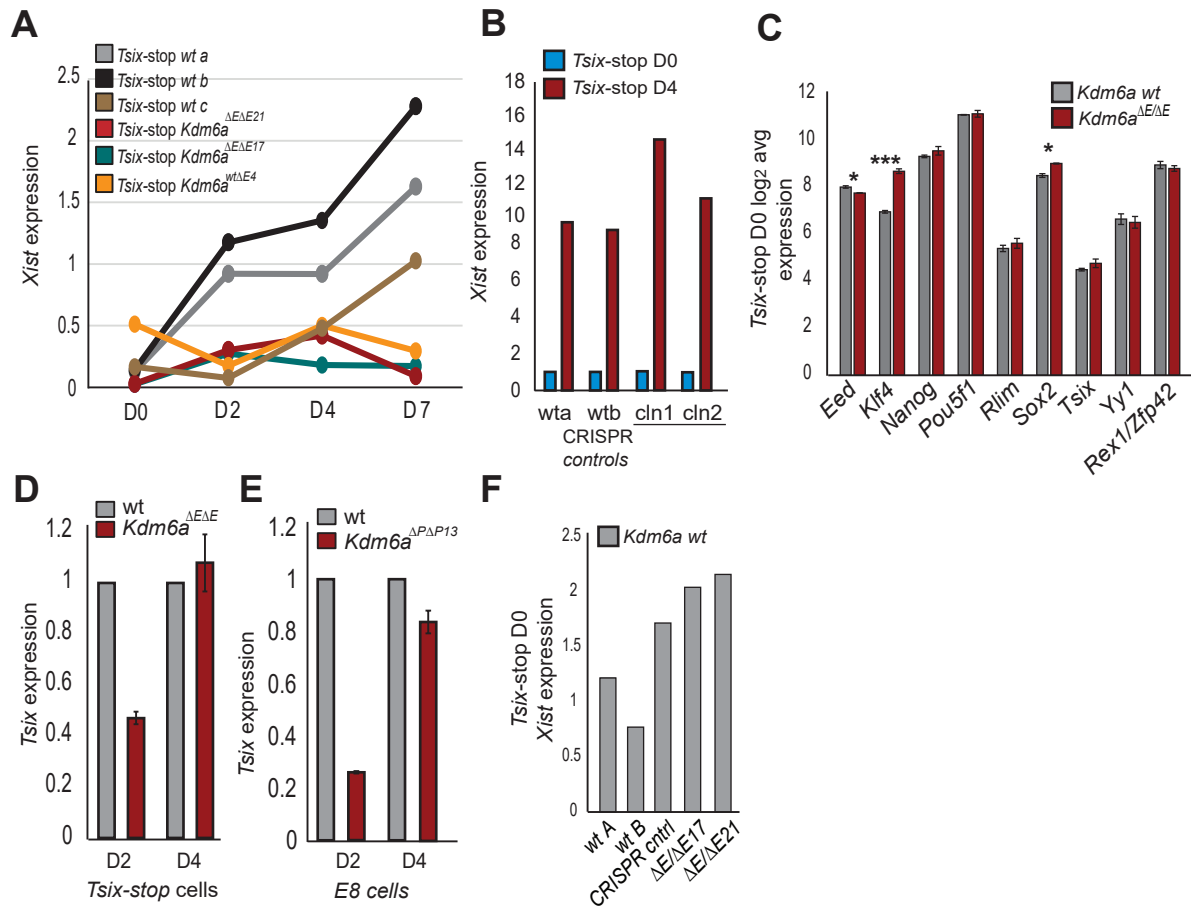

Supplement: Supplementary file 5 — Additional file 5:Supplemental figure S2. Confirmation of Xist expression changes in Kdm6a KO cells. (A) qRT-PCR of Xist expression normalized to Actinβ during ES differentiation in three Tsix-stop wt replicates, the two Kdm6aΔEΔE KO clones, and one Kdm6aΔwtΔEclone. Shown is expression over time for each individual sample. (B) Histogram of qRT-PCR for Xist expression during differentiation in Tsix-stop wt and CRISPR controls (Additional File 1: Table S1). Expression is normalized to Actinβ. Expression at D4 is relative to D0 for each cell line. As expected, Xist is upregulated upon differentiation in wt (wta and wtb replicates) and CRISPR-control clones (cln1 and cln2). (C) Histogram of log2of average TPM values for pluripotency genes and genes known to play a role in Xist repression. Average TPM values are from two Tsix-stop wt and two Kdm6a KO clones (Tsix-Kdm6aΔEΔE17 and Tsix-Kdm6aΔEΔE21). Only Klf4 is called as differentially expressed between wt and KO by DESeq2 (***p<0.002). (D, E) Histograms of qRT-PCR of Tsix expression in (D) Tsix-stop wt and Kdm6aΔEΔE KO clones, and (E) E8 wt and KO clone E8-Kdm6aΔP/ΔP13 at D2 and D4. Expression is normalized to Actinβ and relative to wt. (F) Histogram of average TPM values for Xist in Tsix-stop wt, a CRISPR-control clone (cln1), and two KO clones (Tsix-Kdm6aΔEΔE17, Tsix-Kdm6aΔEΔE21) at D0, confirming that Xist expression is very low in ES cells cultured with serum. [file 13293_2024_683_MOESM5_ESM.pdf]

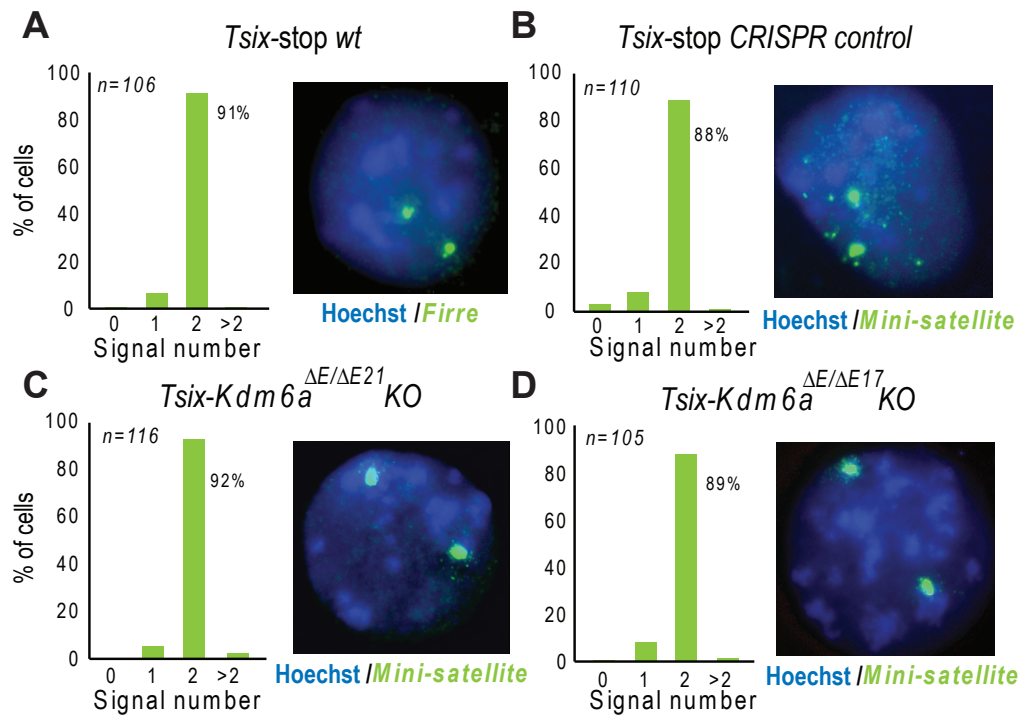

Supplement: Supplementary file 6 — Additional file 6 Supplemental figure S3. Two X chromosomes are present in wt and KO Tsix-stop cells. (A, B) DNA-FISH using probes specific for the X-linked gene Firre or an X-linked mini-satellite repeat region (Dxz4) labelled in green in Tsix-stop wt (A) and CRISPR-control (cln1) (B). Examples of nuclei with two green signals representing the two X chromosomes are shown along with histograms of the number of signals in nuclei scored. n indicates the number of nuclei scored. Nuclei are counterstained with Hoechst 33342. (C, D) Same analysis in as in (A, B), but in the Tsix-stop Kdm6a KO cell clones (Tsix-Kdm6aΔEΔE17 and Tsix-Kdm6aΔEΔE21). [file 13293_2024_683_MOESM6_ESM.pdf]

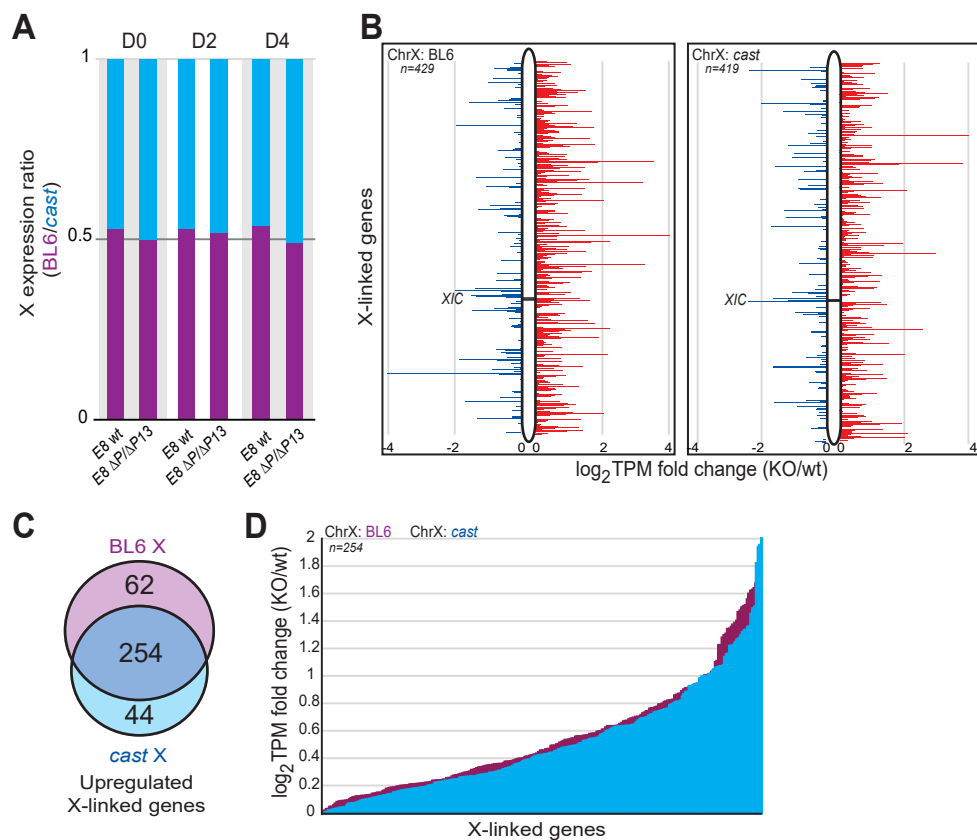

Supplement: Supplementary file 7 — Additional file 7 Supplemental Figure S4. Confirmation of X-linked gene expression changes in E8-Kdm6aΔPΔP13 KO cells. (A) Allelic X-linked gene expression ratios (BL6:cast) in E8 wt and KO cells (E8-Kdm6aΔPΔP13) at D0, D2, and D4 of differentiation. Ratios between expressed X-linked genes (>1TPM) are shown. X expression ratios near 0.5 reflect the largely random nature of XCI in these cells. (B) Plots of expression changes from the BL6 and cast X chromosomes in E8 wt and KO cells (E8-Kdm6aΔPΔP13) at D4. The XIC is highlighted. Log2 TPM fold change (KO/wt) along the X chromosomes is shown. Genes with a decrease of expression in KO versus wt are in blue, and with an increase in red. (D) Venn diagram of the number of X-linked genes with upregulation following Kdm6a KO. The majority of genes are upregulated from the BL6 and cast X chromosomes. (D) Plots of expression changes (based on log2 TPM) for genes with increased expression from each X chromosome (BL6 purple and cast blue) between E8 wt and E8-Kdm6aΔPΔP13 KO cells at D4. The X-axis includes 254 X-linked genes. There is a slightly greater increase in expression from the BL6 X chromosome (purple). [file 13293_2024_683_MOESM7_ESM.pdf]

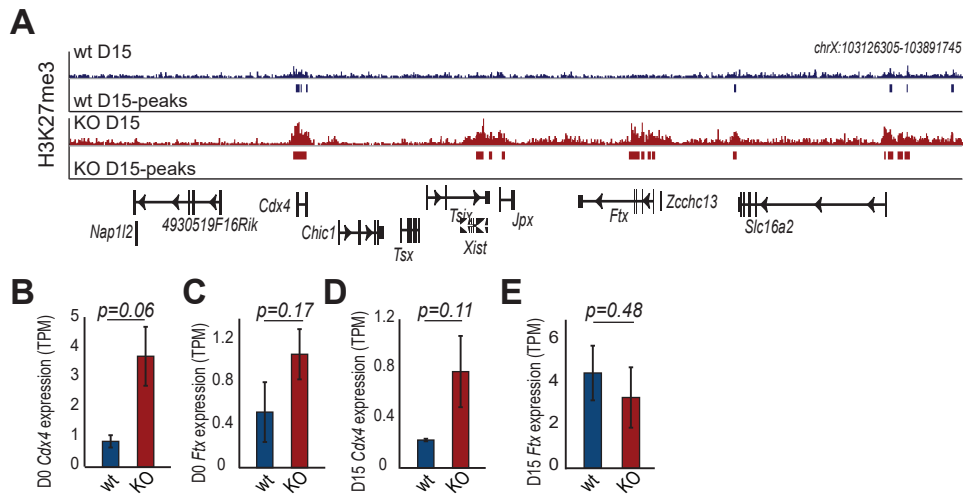

Supplement: Supplementary file 10 — Additional file 10 Supplemental figure S5. H3K27me3 profiles along the XIC in wt and Kdm6a KO cells. (A) IGV browser view of profiles and peaks of H3K27me3 enrichment at the XIC (chrX: 103126305-103891745) show increased H3K27me3 at Cdx4, Xist, and Ftx in differentiated Kdm6a KO cells. Tsix-stop wt (blue) and KO clone Tsix-Kdm6aΔEΔE17 (red). (B, C) Histograms of expression (TPM) of Cdx4 (B) and Ftx (C) in D0 Tsix-stop wt (blue) and Kdm6a KO clones (red). Both wt and KO ES cells show low expression of Cdx4 and Ftx (~1.5TPM or less). (D, E) Same analysis as in (B) and (C) but for D15. Both wt and KO ES cells show low expression of Cdx4 and Ftx (~1TPM or less). [file 13293_2024_683_MOESM10_ESM.pdf]

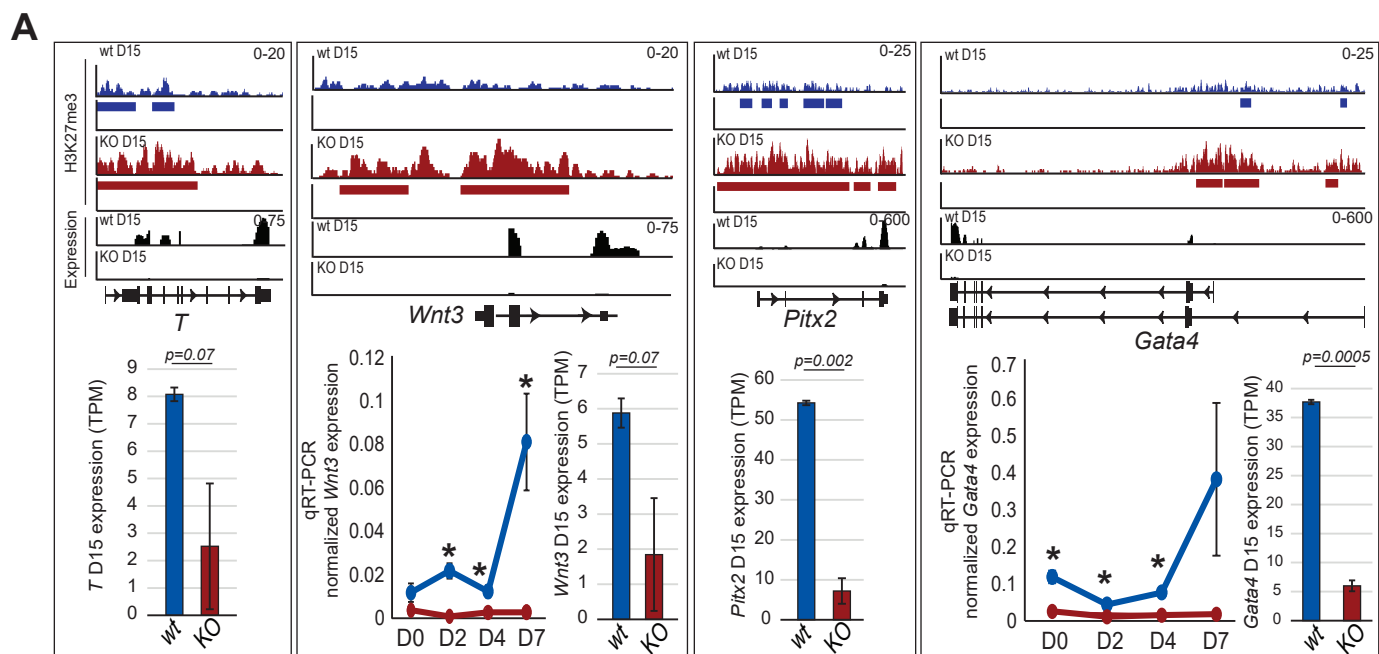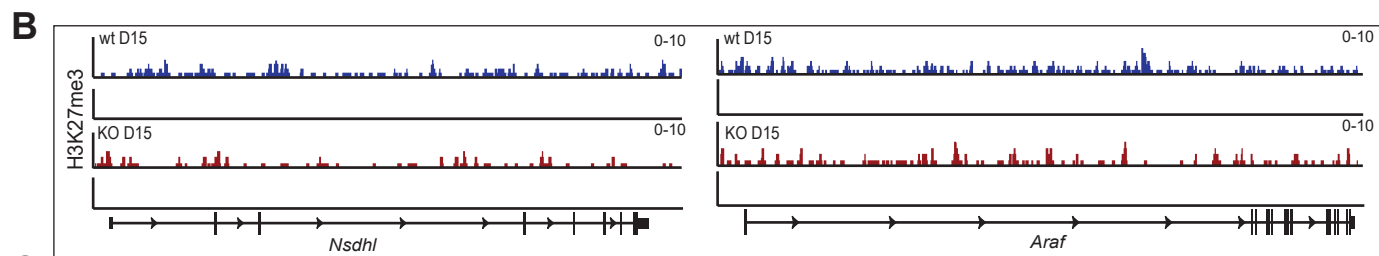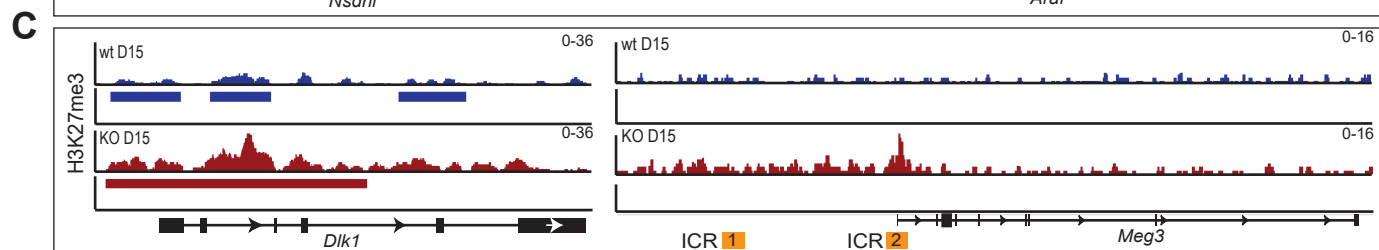

Supplement: Supplementary file 11 — Additional file 11 Supplemental figure S6. Epigenetic and expression changes at known KDM6A target genes. (A) IGV browser views of profiles of H3K27me3 enrichment and peaks at known KDM6A target genes in differentiated Tsix-stop wt cells (blue) and KO clones (Tsix-Kdm6aΔEΔE17) (red). Peaks are represented by colored bars below the signal profiles. Below are RNA-seq expression profiles (black) in wt and KO cells. Histograms show TPM expression values for T and Pitx2 in two differentiated wt clones and KO clones. For Wnt3 and Gata4, both changes in expression at D0-7 obtained by qRT-PCR during differentiation and histograms of TPM values following differentiation are shown (*p<0.05). KO represents an average for the Tsix-Kdm6aΔEΔE17 and Tsix-Kdm6aΔEΔE21 clones. Values are normalized to Actinβ. (B) Same analysis as in (A), but for Nsdhl and Araf, two genes that do not show significant downregulation following Kdm6a KO. (C) Same analysis as in (A), but at the Dlk1/Meg3 imprinted locus at D15 where H3K27me3 markedly increases at the promoter of Meg3 in KO cells, including at the imprinting control region ICR (2) and the intergenic ICR (1). The scales of the profiles shown in (A), (B) and (C) are indicated in the upper right corners. [file 13293_2024_683_MOESM11_ESM.zip › 13293_2024_683_MOESM11_ESM/1-Additional file 11_S6.pdf]

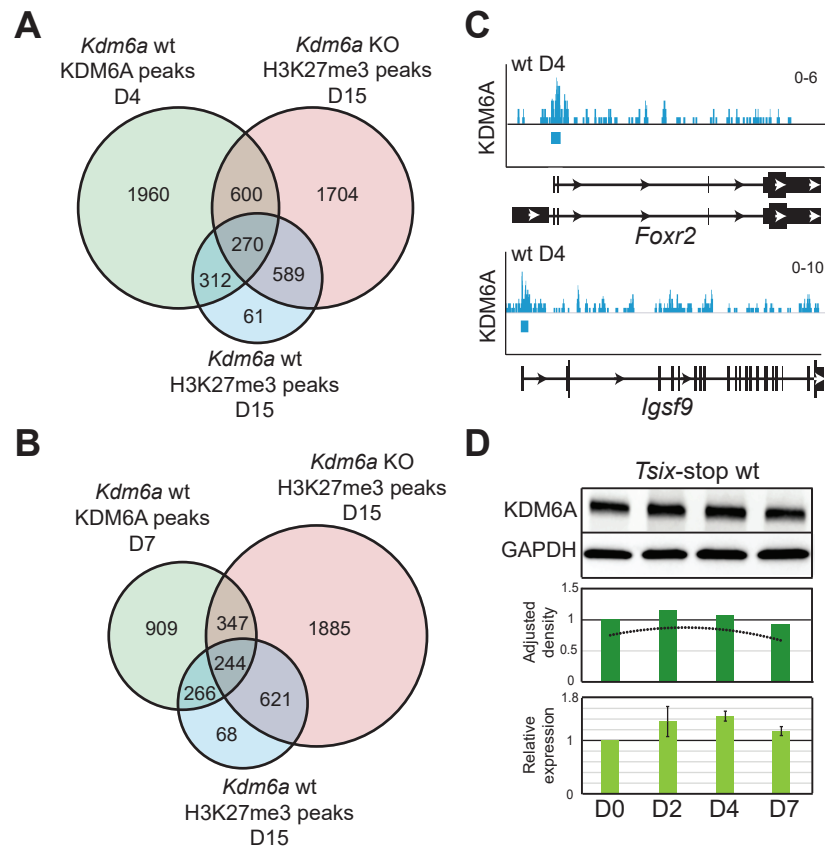

Supplement: Supplementary file 13 — Additional file 13 Supplemental figure S7. Characterization of KDM6A binding, expression and protein levels during differentiation of Tsix-stop cells. (A) Venn diagram comparing number of peaks of KDM6A in wt Tsix-stop cells at D4 of differentiation to H3K27me3 peaks in wt and Kdm6a KO cells at D15 of differentiation. (B) Same analysis as in (A) but using KDM6A binding at D7 for comparisons. (C) KDM6A enrichment profiles at Foxr2 and Igsf9, two genes known to be regulated by KDM6A. D4 signal and peaks in wt Tsix-stop cells are shown. Scales are shown in the upper right corner. (D) Top, protein blots of KDM6A in wt Tsix-stop (top) during differentiation. Adjusted density was calculated by normalizing levels to the GAPDH loading control using ImageJ (middle). Bottom, histograms of qRT-PCR of Kdm6a expression in two wt Tsix-stop replicates during differentiation. Expression was normalized using Actinβ and expression relative to D0 is shown. [file 13293_2024_683_MOESM13_ESM.pdf]

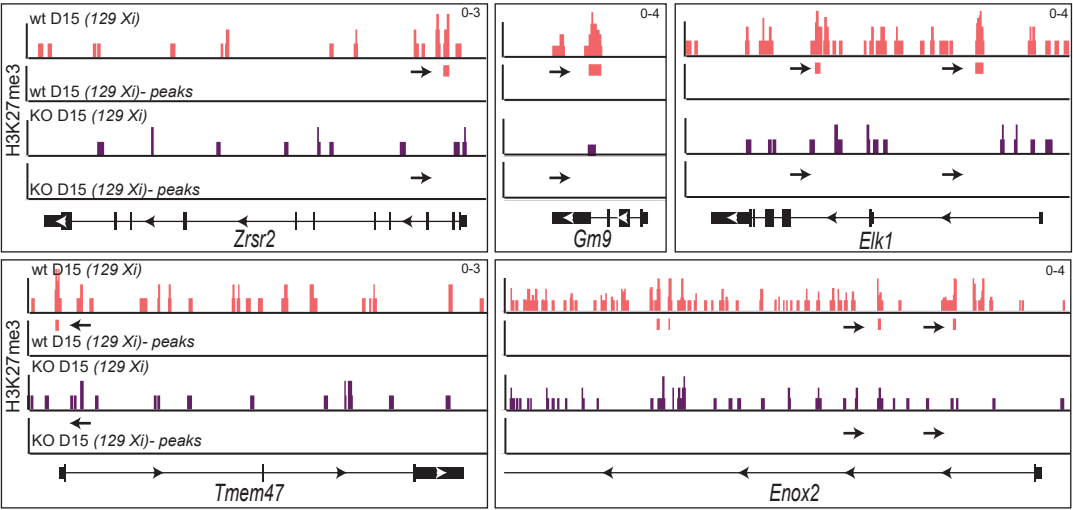

Supplement: Supplementary file 15 — Additional file 15: Supplemental figure S8. Allelic H3K27me3 enrichment at X-linked genes with Xi-specific expression increases in Kdm6a KO Tsix-stop cells. IGV browser views of Xi H3K27me3 signal and peaks at a subset of genes with increased expression from the Xi in Kdm6a KO cells. Wt cells are in pink and KO cells are in purple. Some genes with Xi-specific increases in expression following differentiation (D15) show lack of H3K27me3 near their TSS, due to failure of XCI and recruitment of PRC complexes. [file 13293_2024_683_MOESM15_ESM.pdf]
